# Health-related quality of life and associated factors among patients with diabetes mellitus at the University of Gondar referral hospital

**DOI:** 10.1186/s12955-020-01311-5

**Published:** 2020-03-10

**Authors:** Andualem Yalew Aschalew, Mezgebu Yitayal, Amare Minyihun

**Affiliations:** Department of Health Systems and Policy, Institute of Public Health, College of Medicine and Health Sciences, University of Gondar, Gondar, Ethiopia

**Keywords:** Diabetes mellitus, Health-related quality of life, WHOQOL-BREF, Ethiopia

## Abstract

**Background:**

Diabetes mellitus, which has a wide range of effects on the physical, social and psychological aspects of the well-being of a person, is a common and challenging chronic disease that causes a significant rate of morbidity and mortality. However, studies in our country, by and large, focused on the impact of the disease in terms of mortality and morbidity alone. Therefore, the objective of this study was to assess the health-related quality of life (HRQOL) and associated factors of diabetic patients at the University of Gondar referral hospital, Ethiopia.

**Methods:**

A facility-based cross-sectional study was conducted at the University of Gondar referral hospital from April to May 2017. A generic World Health Organization Quality of Life (WHOQOL-BREF) questionnaire was used to measure the HRQOL. The data were analyzed by Stata version 12. Multiple Linear Regression analysis with *P*-value 0.05 was used to measure the degree of association between HRQOL and independent variables.

**Results:**

A total of 408 patients with Diabetes Mellitus were included in the study. The HRQOL scores for physical, psychological, social and environmental domains were 50.9, 54.5, 55.8 and 47.3, respectively. Diabetes-related complications had a significant association with all except the psychological domain. Higher HRQOL was associated with exercising, following the recommended diet, foot care, sensible drinking and the absence of co-morbidities. However, old age, unemployment and being single and widower had a significant association with lower HRQOL.

**Conclusion:**

The environmental and physical domains of HRQOL scores were the lowest compared to the social and psychological domains. Old age and living in rural area had a significant association with a lower HRQOL, whereas the absence of diabetes-related complications, exercising, general diet and foot care had a significant association with better HRQOL of patients. Therefore, strong advice on the recommended lifestyle is important, and old patients and rural dwellers should get due attention. In addition, the prevention of diabetes-related complications is important to improve the patient HRQOL which is an important outcome measurement from the patient’s perspective related to the impact of the disease. Therefore, including HRQOL assessment as part of routine management is necessary.

## Background

Diabetes mellitus (DM) is one of the chronic diseases that affect both developed and developing countries. The International Diabetics Federation (IDF) reported that in 2015, the disease affected 415 million people worldwide and will rise to 642 million in 2040. An estimated 14.2 million adults aged 20–79 suffer from diabetes in the Sub-saharan Africa. Ethiopia is one of the most populous countries in this region and has the highest number (1.3 million) of people with diabetes. The prevalence of DM, which is one of the four major chronic diseases in the country, is about 3.8% [1, 2].

The impact of diabetes on a patient can be measured by traditional methods, like biochemical, morbidity and mortality although attention has recently been given to measuring health-related quality of life (HRQOL). HRQOL is important to assess the impact of the disease from the patient’s perspective [3–8]. Hence, HRQOL can be defined as “the subjective assessment of the impact of disease and its treatment across the physical, psychological and social domains of functioning and well-being” [9].

Diabetic patients feel about their blood glucose level and worry about the complications they might be developing or actually exist. Moreover, the never-ending care and lifestyle adjustments, like dietary change and exercise have an impact on patients’ HRQOL (physical, emotional and social well-being) [10, 11]. Different studies have shown that the presence of diabetes has an impact on HRQOL and reduces the physical, psychological, environmental and social domains of health [12–15]. People with diabetes experience significant impairment in their HRQOL compared to non-diabetes people [16–18].

Health professionals can identify the physiological derangement and degree of deteriorations due to diabetes. Nevertheless, an individual patient’s health perceptions and well-being are not directly proportional to symptoms and functional limitations which in turn are not directly proportional to physiological and anatomic abnormalities. Therefore, the effects flowing from biological abnormalities to HRQOL are mediated and modified by psychological, social and cultural factors [19]. However, studies in our country, including those in the current study area focus on the impact of diabetes in terms of morbidity and mortality alone [20–22]. As far as we know, there has been no study on the psychosocial impact of diabetes in the study area. Therefore, this study aimed to determine the HRQOL of diabetic patients and to identify factors associated with it.

## Methods

### Study design and setting

An institution-based cross-sectional study was conducted at the University of Gondar referral hospital chronic illness follow-up outpatient clinic from April to May 2017 to assess HRQOL. The hospital, located in North Gondar zone, is one of the tertiary level health care facilities in Ethiopia. It has an outpatient department for chronic illness follow up and diabetes treatment provided 2 days a week to an average of 900 diabetic patients per month. It also has inpatient facilities where medical care is provided throughout the week.

### Study population and sampling procedures

The population was all adult diabetic patients in the chronic illness follow up clinic during the study. All adult diabetic patients on follow-up for at least 6 months were included, whereas individuals with gestational diabetes and patients who were unable to communicate were excluded. The sample size was determined using a power approach (double population formula) from a previous study [23] by considering type I error of 0.05, type II error of 0.10, confidence interval 95%, standard deviation (SD) one 17.22, SD two 13.79, mean difference 5.2 and non-response 10%. Therefore, the final sample size of the study was 416. All diabetic patients who came to the University of Gondar referral hospital for follow ups were recruited consecutively until the minimum required sample size was reached.

### Data collection tools and procedures

The World Health Organization quality of life (WHOQOL-BREF) the short version of the WHOQOL-100 SCALE was used to collect data. The questionnaire which contains 26 items was developed with 15 international field centers to obtain an assessment tool applicable cross-culturally [24]. The WHOQOL-BREF contains four specific domains (physical, psychological, social and environmental). The questionnaire was first adopted in the English language and translated to Amharic and back-translated to English to maintain its consistency. Factors relating to socio-demographics (age, sex, marital status, educational level, occupation, residence, ethnicity, religion and wealth index), clinical data (duration of diabetes, type of diabetes, diabetes-related complications, co-morbidities: any chronic diseases other than diabetes mellitus, body mass index, type of treatment and fasting blood glucose) and Lifestyle (smoking, physical exercise, diet, foot care and alcohol consumption) were included in the questionnaire.

Data on patient socio-demographics, lifestyle, HRQOL and some clinical data were collected by a trained interviewer, while some clinical data (co-morbidities, diabetes-related complications, diabetes type and fasting blood sugar) were taken from patients’ medical record cards.

### Operational definitions

#### Health-related quality of life

The instrument used was the WHOQOL-BREF of the WHOQOL-100 scale. This questionnaire contains 26 items computed into four specific domains: physical, psychological, social and environmental. The mean score on items within each domain was used to calculate the domains score. Higher scores denoted a higher HRQOL, and lower scores indicated lower HRQOL [24].

#### Body mass index

BMI was calculated by dividing weight into height squared and divided into four categories based on WHO classification [25]: underweight = < 18.5, normal weight = 18.5–24.9, overweight = 25–29.9 and obese = ≥30.

#### Alcohol

Alcohol consumption was assessed by using Fast alcohol screening test and split into two categories [26]: Non-hazardous drinker = < 3 and Hazardous drinker = ≥ 3.

#### Summary diabetes self-care activity (SDSCA)

This tool assessed the number of days per week on a scale of 0–7 on which the patient performed the recommended self-care activities [27].

General diet = Mean number of days the patient follows the recommended diet plan.

Specific diet = Mean number of days the patient eats fruits and fatty foods.

Exercise = Mean number of days the patient performs a minimum of 30 min activity.

Foot care = Mean number of days the patient takes care of their feet and check the inner part of their shoes.

Smoking status = (1 = smoker 2 = non-smoker).

### Data analysis

The collected data were checked for completeness. Then, codes were given to each question and entered into Epi-Info Version 7 Software. Further analysis was done with Stata version 12. Where an item was missing, the mean of other items in the domain was substituted. But when more than two items were missing from the domain, the domain score was not calculated with the exception of domain 3, where the domain should be calculated if only one item is missing. Negatively framed questions (items 3, 4 and 26) were transformed into positively framed ones. Cronbach alpha was used to check the reliability of the items and domains. Raw and transformed scores were considered for the outcome variables. Summary statistics were done for the outcome and independent variables. Model assumptions (normality, equal variance, multicollinearity and linearity) were checked. Simple linear regression analysis was done to identify factors associated with each domain of HRQOL independently at a *P*-value < 0.2. Variables that were significant at a *p*-value of < 0.2 were selected for the final multiple linear regression model. In the multiple linear regression analysis, variables with *P*-values of < 0.05 were considered statistically significant.

## Results

### Socio-demographic and economic characteristics of the study participants

A total of 416 diabetic patients participated in the study. Eight (1.92%) questionnaires were excluded from the analysis because they were incomplete. Of the participants, 54.7% were male, and 33.7% were unable to read and write. Their mean age (SD) was 47.48 ± 14.9 years (Table 1).

**Table 1:** Socio-demographic and economic characteristics of study participants (*n* = 408)

Variables	Description	Frequency (%)	Mean ± SD
Sex	Male	223 (54.66)	
	Female	185 (45.34)	
Age	Age in years		47.48 ± 14.9
Marital status	Single	60 (14.7)	
	Married	241 (59.1)	
	Windowed	54 (13.2)	
	Divorced	53 (13.0)	
Occupation	Unemployed	19 (4.66)	
	Farmer	92 (22.55)	
	Retired	25 (6.13)	
	Employed	152 (37.25)	
	Housewife	103 (25.25)	
	Student	17 (4.16)	
Educational	Unable to read and write	137 (33.58)	
	Primary	154 (37.75)	
	Secondary	34 (8.33)	
	Above Secondary	83 (20.34)	
Residence	Urban	285 (69.85)	
	Rural	123 (30.15)	
Wealth quintile	Poor (1st quintile)	138 (33.82)	
	Medium (2nd quintile)	138 (33.82)	
	Rich (3rd quintile)	132 (32.36)	

*SD* Standard deviation

### Clinical and lifestyle characteristics of study participants

Approximately, 56.6% of the participants were type 2 diabetes; 28.92% had co-morbidities, and 21.57% developed a diabetes-related complication (Table 2).

**Table 2:** Clinical and lifestyle characteristics of study participants (*n* = 408)

Variables	Description	Frequency (%)	Mean ± SD
Diabetes type	Type 1	177 (43.38)	
	Type 2	231 (56.62)	
Duration of disease	Duration in years		6.82 ± 4.55
Fasting blood sugar	< 140	135 (33.09)	
	≥140	273 (66.91)	
Type of treatment	Oral medication	143 (35.05)	
	Injection	233 (57.11)	
	Both	32 (7.84)	
Co-morbidities	Present	118 (28.92)	
	Absent	290 (71.08)	
DRC	Present	88 (21.57)	
	Absent	320 (78.43)	
BMI	Under weight	26 (6.37)	
	Normal weight	241 (59.07)	
	Overweight	108 (26.47)	
	Obese	33 (8.09)	
Alcohol	Hazardous drinker	39 (18.06)	
	Sensible drinker	177 (81.94)	
SDSCA	General diet		2.75 ± 1.99
	Specific diet		3.31 ± 1.01
	Exercise		2.07 ± 1.75
	Foot care		4.27 ± 2.07
	Smoker	17 (4.17)	
	Nonsmoker	391 (95.83)	

*BMI* Body mass index, *DRC* Diabetes-related complication, *SDSCA* Summary of diabetes self-care activities, *SD* Standard deviation

### Health-related quality of life

In this study, 41.91% of the participants rated their quality of life as good, and 18.14% were satisfied with their current health status (Table 3).

**Table 3:** Self-rating of HRQOL and health status satisfaction of the participants (*n* = 408)

Self-rating of HRQOL		Satisfaction with health status	
Response	Frequency (%)	Response	Frequency (%)
Very poor	5 (1.23)	Very dissatisfied	13 (3.19)
Poor	89 (21.81)	Dissatisfied	86 (21.08)
Neutral	137 (33.58)	Neutral	226 (55.39)
Good	171 (41.91)	Satisfied	74 (18.14)
Very good	6 (1.1)	Very satisfied	9 (2.21)
Total	408 (100)	Total	408 (100)

The four domains had good internal reliability with Cronbach Alpha: physical α = 0.77, psychological α = 0.69, social α = 0.73 and environmental α = 0.71. out of the 4 domains, the environmental domain HRQOL had the lowest mean score. In contrast, the social domain of HRQOL had the highest score (Table 4).

**Table 4:** HRQOL domain score of study participants (*n* = 408)

Domains	n	Mean ± SD	95% CI
Physical	408	50.97 ± 13.89	49.62–52.32
Psychological	408	54.55 ± 13.36	53.25–55.85
Social	408	55.88 ± 17.63	54.16–57.59
Environmental	408	47.31 ± 12.51	46.10–48.53

*CI* Confidence interval, *SD* Standard deviation

### Factors associated with health-related quality of life

In this study, some socio-demographic, clinical and lifestyle variables were statistically significant determinants of each domain of HRQOL at a *p*-value of 0.05.

Age had a significant association (B = −.13, 95% CI = − 2.5, − 0.1), (B = −.16, 95% CI = −.30, −.01) and (B = −.20, 95% CI = −.34, −.05) with physical, psychological and social domains, respectively. Being single (B = − 6.70, 95% CI = − 12.43, −.97) and being widowed (B = − 6.64, 95% CI = − 12.6, -.613) had a significan association with the social and psychological domains, respectively. The environmental domain was significantly associated with secondary and above education level (B = 3.87, 95% CI = .14, 7.60), residence (B = − 3.88, 95% CI = − 7.72, −.04) and foot care (B = 1.12, 95% CI = .55, 1.70). Co-morbidity and occupation had a significant associate with physical domaim (B = 5.55, 95% CI = 2.55, 8.55). Diabetes-related complication statisticaly associated (B = 4.5, 95% CI = 1.17, 7.83), (B = 7.69, 95% CI = 3.18, 12.2) and (B = 3.88, 95% CI = .99, 6.77) with physical, social and environmental domains, respectively. Exercise was associated (B = 0.89, 95% CI = .08, 1.69) and (B = 1.28, 95% CI = .28, 2.29) with the physical and psychlogical domains, respectively. General diet had also a significant association (B = 0.84, 95% CI = 07, 1.61), (B = 1.14, 95% CI = .08, 2.20) and (B = 1.41, 95% CI = .71, 2.10) with the physical, psychlogical and environmental domains, respectively. Being hazardous drinker was statistically associate (B = 8.14, 95% CI = 4.08, 12.2) with the psychologicla domain (Table 5).

**Table 5:** Factors associated with the four domains of HRQOL of diabetic patients

Variables	Physical		Psychological		Social		Environmental	
	ß	95% CI	ß	95% CI	ß	95% CI	ß	95% CI
Age	−.13	−2.5, −0.1*	−.16	−.30, −.01*	−.20	−.34, −.05**		
Marital status								
Married			–	–	–	–		
Divorce			−2.87	−7.92, 2.18	− 3.14	−8.33, 2.04		
Single			−4.12	−8.82, .57	−6.70	−12.43, −.97*		
widowed			− 6.64	−12.6, -.613*	−.60	−6.01, 4.81		
Occupation								
Unemployed	–	–						
Farmer	8.33	1.75, 14.90*						
Retired	10.7	2.92, 8.54**						
Employed	7.12	.97, 13.27*						
Housewife	5.88	−.62, 2.40						
Student	9.50	.90, 18.09*						
Education								
Unable to read & write							–	–
Primary							1.47	−1.24, 4.19
Secondary							1.16	−3.35, 5.67
Above secondary							3.87	.14, 7.60*
Residence								
Urban							–	–
Rural							−3.88	−7.72, −.04*
Co-morbidities								
Yes	–	–						
No	5.5	2.55, 8.55***						
DRC								
Yes	–	–			–	–	–	–
No	4.5	1.17, 7.83**			7.69	3.18, 12.2**	3.88	.99, 6.77**
Exercise	.89	.08, 1.69*	1.28	.28, 2.29*				
General diet	.84	.07, 1.61*	1.14	.08, 2.20*			1.41	.71, 2.10***
Foot care							1.12	.55, 1.70***
Alcohol								
Hazardous drinker			–	–				
Sensible drinker			8.14	4.08, 12.2***				

*DRC* Diabetes-related complication, * variables significant with *p*-value ≤0.05, ** variables significant with *p*-value ≤0.01, ***Variables significant with *p*-value ≤0.0001

## Discussion

This study was done on patients with DM at the University of Gondar referral hospital. The results revealed that diabetes had an impact on HRQOL for diabetic patients in different domains. The maximum and minimum scores were related to social and environmental domains, respectively. Age, general diet and diabetes-related complications had a significant association with at least three domains of HRQOL.

The study found that patients had the lowest score (47.31 ± 2.51 out of 100) in the environmental domain compared to the three domains, whereas the social domain had the highest score (55.88 ± 17.63). The psychological and physical domains were also approximately average out of hundred. Although there was no cut-off point for WHOQOL-BREF to categorize HRQOL as high or low, the finding showed the score of each domain was approximately average and diabetes had an impact on patients’ health and well-being. Lifestyle modifications of diabetes treatment such, as diet (eating carefully), exercising, monitoring blood glucose, worry about complications associated with diabetes and the dependence of life (daily activity) on medication are some of the explanations for the reduction of HRQOL. These might cause negative feelings, such as depression, affect social interactions and recreational activities. Different studies have also shown that diabetes affects patient’s HRQOL compared to healthy individuals [28, 29]. The result is in line with that of a study conducted in Kenya [12] in terms of the sequence of domains affected by diabetes.

In terms of all domains, the HRQOL score of this study is higher than that of Palestine, Gaza, which used similar tools [17]. The Possible explanation might be differences in psycho-social, cultural, economic, and environmental conditions. For instance, the participants of the present study lived in a stable and peaceful environment and had relatively their own living facility, access to the health facilities and other infrastructures compared to refugee patients in Gaza who depended on refugee camp supplies. Studies in Benin, Nigeria and Uganda [28, 30] also have lower scores than the current study. A possible explanation might be differences in measurement tools.

A study in Iran with WHOQOL-BREF [23] shows the four domains of HRQOL are higher than those of the current study. A possible explanation might be differences in economic status, satisfaction with the infrastructure and health care service and clinical characteristics of patients. Moreover, studies from India also have higher scores than the current study [31–33].

Age had a significant association with all domains of HRQOL except the environmental domain. This is in line with those of studies in Kenya and Singapore [12, 34]. This can be explained by the fact that age is related to several changes in the body and increases the risk of developing co-morbid diseases and further reduces individual well-being. The American Diabetes Association [35] also shows that the aging process leads to a degeneration of muscles, ligaments, bones, and joints and that diabetes may exacerbate the problem. Moreover, occupation and education had a significant association with the physical and environmental domains of HRQOL, respectively. Employed, farmer and retired had a higher score on the physical domain compared to unemployed. Patients with above secondary education level had a higher score on the environmental domain than those who were unable to read and write [13, 30, 36]. Education is an essential factor in understanding self-care management and perception of self-worth. The patients with a high educational level can easily read and understand the effects of diabetes and this might lead to better awareness about the disease such as complications. Furthermore, it contributes to a high rate of adherence to self-care management such as diet.

This study showed that patients without co-morbidities had a better score in the physical domains of HRQOL than patients with co-morbidities. This is supported by studies in Nigeria and Singapore [34, 37]. Moreover, diabetic patients without diabetes-related complications had a better HRQOL in all domains except the psychological domain. This is in line with the findings of in Palestine and Singapore [17, 34] that patients with diabetes-related complications had a lower HRQOL.

Marital status had a significant association with the social domain. Those who were single were more likely to have a lower HRQOL compared to the married ones. This is supported by a study in Nigeria, which reported that singles had lower odds of HRQOL than couples [38]. In addition, compared to married women widowed women had a lower score of the psychological domain of HRQOL. This might be because the probability of getting social or relative support is better for those who live in marital bonds.

Out of the lifestyle factors, exercise had a significant association with physical and psychological domains. Following a recommended general diet also had a significant association with all domains except the social domain. An interventional study in Sandiego, California [39], showed that exercising and adhering to the recommended diet had a positive impact on the HRQOL of patients. Studies in Nigeria and Canada [36, 37] are also in line with this finding. As the number of days of foot care increased the psychological and environmental domains of HRQOL also improved. A study done in Uganda [30] revealed that patients with foot ulcers had a low HRQOL. Hence, foot care was a good measure to prevent foot ulcer and improve HRQOL by increasing patients’ sense of physical safety, enabling them to participate in recreation, and avoiding long-treatment, hospitalization and amputation. Finally, sensible drinkers had a better HRQOL of the psychological domain compared to the hazardous drinkers. Studies showed that moderate to heavy drinkers had a lower HRQOL (mental health) than nondrinkers or occasionally drinkers [40, 41]. Alcohol consumption can impair an individual’s cognitive and altered consciousness. Studies revealed that alcohol consumption (excessive) impaired glycemic control which leads to worrying about the level of glucose, depression, complications and reduces satisfaction with their health status [42].

Compared to urban residents rural dwellers had a lower score in the environmental domain of HRQOL. Although evidence that directly compares residence with HRQOL is limited, there are clear differences with respect to access to health services, information, education and living standards between rural and urban settings. All these might contribute to the low score of the environmental domain of HRQOL. Studies, from the normal population, showed that subjects who live in the urban areas had a higher HRQOL than subjects who live in rural areas [43, 44].

### Limitation of the study

This was a cross-sectional study which was able only to detect associations, but not causalities. In addition, some important variables, like lipid profile and HgA1c were not included. As the study was conducted in one setting, findings might not be representative of the diabetic patients in other settings.

## Conclusion

Diabetes has an impact on the patient’s HRQOL. The diabetic patients have often expressed their dissatisfaction with their health status and rated their quality of life as “poor” showed that the disease had a marked impact on their HRQOL. Environmental and physical domains of HRQOL were the lowest compared to the social and physical domains. Old age and living in rural areas had a significant association with low HRQOL, whereas the absence of diabetes-related complications, exercise, general diet and foot care were significantly associated with high HRQOL. Therefore, providing strong advice on the recommended lifestyle is important, and old age and rural dweller patients should get emphasis. In this respect, the prevention of diabetes-related complications is important to improve patient’s HRQOL which is an important outcome measurement from the patient’s perspective relating to the impact of the disease. Therefore, including HRQOL assessment as part of routine management is necessary. Since HRQOL is multidimensional, establishment of a multidisciplinary team of physicians, nutritionists, fitness coaches and social workers is important that works to educate and empower patients. Finally, a further longitudinal study will be needed for understanding the associations of factors influencing HRQOL.

## Data Availability

The datasets supporting the conclusions of this article are available upon request to the corresponding author. Due to data protection restrictions and participant confidentiality, we do not make participants’ data publicly available.

## Abbreviation

BMI: Body mass index
CI: Confidence interval
DM: Diabetes Mellitus
DRC: Diabetes-related complication
HRQOL: Health-related quality of life
IDF: International Diabetes Federation
SD: Standard deviation
SDSCA: Summary of diabetes self-care activities
WHO: World Health Organization
WHOQOL-BREF: World health organization quality of life bref
